# Assessing inter- and intra-examiner reliability of orthodontists in devising incisor position objectives on cephalograms: a comparative study between senior and junior practitioners

**DOI:** 10.1186/s12903-023-03638-z

**Published:** 2023-12-01

**Authors:** Xue Li, Zhenxing Tang, Yu Li, Xiaowen Niu, Fang Zhou

**Affiliations:** 1 Department of Orthodontics, School of Stomatology, Xi’an Medical University, Xi’an, 710021 China; 2 State Key Laboratory of Oral Diseases & National Clinical Research Center for Oral Diseases & Department of Orthodontics, West China Hospital of Stomatology, Sichuan University, Chengdu, 610041 China; 3 Department of Dentistry and Oral Health, Aarhus University, Aarhus, 8000 Denmark

**Keywords:** Orthodontic treatment planning, Cephalograms, Incisor position objectives, Intra-examiner reliability, Inter-examiner reliability

## Abstract

**Background:**

Effective orthodontic treatment planning hinges on accurately defining incisor position objectives (IPO) in cephalograms. The purpose of this study was to estimate the inter-examiner and intra-examiner reliability of different orthodontists in devising IPOs on cephalograms.

**Methods:**

Ten orthodontists, who were divided into to the senior group (N = 5) and the junior group (N = 5) based on their clinical experience, formulated IPOs for 60 pre-treatment cephalograms twice with an interval of 2 weeks, utilizing SmartOrtho software. The type and magnitude of movement were read directly in the software. A paired *t*-test assessed the absolute differences between the first and second IPO devising within each group and between the senior and junior groups in each time’s IPO devising. The intra-examiner and inter-examiner reliabilities were calculated.

**Results:**

There were significant differences in all types of upper incisor movement and lower incisor protrusion/retraction movement between the first and second IPO devising of the senior group. The junior group exhibited significant differences in the twice the upper incisor extrusion/intrusion movement and upper incisor torque movement devising. Additionally, significant differences in all types of incisor movement between the senior and junior groups in each time’s IPO devising. Intra-examiner reliabilities were excellent for both two groups and moderate for the junior group in most types of incisor movement, respectively. The inter-examiner reliability between the two groups ranged from moderate to good across different types of incisor movement.

**Conclusions:**

Among orthodontists, both senior and junior practitioners displayed the best inter-examiner reliability in lower incisor extrusion/intrusion movement. In terms of intra-examiner reliability, senior orthodontists had better intra-examiner reliability in upper incisor position objectives devising than the junior orthodontists. Furthermore, senior orthodontists tended to adopt a more recessive, intrusive, and lingually torqued incisor position approach compared to junior orthodontists.

## Background

The position of incisors is important for dentofacial aesthetics and orthodontic treatment planning. A number of cephalometric analyses put a significant weight on incisors, for example the Downs analysis [1], the Tweed triangle [2], and McNamara analysis [3]. Among these, the Tweed triangle focuses on evaluating the position of lower incisors. Charles Tweed emphasized the significant role of the Frankfort mandibular incisor angle (FMIA) in facial aesthetics and should be varied according to Frankfort mandibular angle (FMA) [4]. However, the normal parameters of FMIA have been found to vary among different racial and gender groups [4–6], which limits its applicability. Cecil Steiner thought that compromises of incisors must be made to successfully camouflage the discrepancy of jaw relationship. He proposed a series of “acceptable arrangement” values to guide incisor arrangement for different ANB angle [7]. According to his acceptable compromises, the linear and angular values of the maxillary and mandibular incisors should be changed with the ANB angles. However, it has been revealed that the data of so-called acceptable arrangements stem from a geometric scheme rather than being derived from a comprehensive survey of individuals, thus failing to reflect true biologic camouflage [8]. Hence, it is deemed insufficient for precise incisor positioning to use Steiner’s acceptable arrangements as the only reference to devise the incisors position. In contrast, Will Alan Andrews found that populations with pleasing profiles have their maxillary central incisors positioned between the glabella and the FFA point (the midpoint between trichion and glabella for foreheads with flat contour or the midpoint between superion and glabella for foreheads with rounded or angular contour). He suggested that the forehead be used as a landmark of anteroposterior positioning of maxillary incisors to improve facial harmony [9]. This finding provides a new perspective to evaluate the anteroposterior position of maxillary incisor and acts as a supplement tool to cephalometric analysis and repose soft tissue analysis. However, it is a guidance aiming to improving profile aesthetics, regardless of function, stability and safety. Researchers have found that the incidence of lingual alveolar bone defects, such as dehiscence and fenestration, significantly increased in anterior teeth after orthodontic treatment [10], especially in four premolars extracting cases [11]. Bone defects were associated with corresponding reduction in alveolar bone thickness after tooth movement [10]. Besides the risk of bone defect, root resorption is another common detrimental side effect, and the risk is highly related to the direction of tooth movement [12] and the amount of apical displacement [13]. In order to prevent side effects, the IPO must be carefully and precisely devised and controlled within the physiological range, according to each patient’s anatomic structure. With the evolution of customized orthodontic appliances, teeth can be moved exactly the same as digital arrangement designed [14–17], calling for accurate designs of teeth movement from the very beginning of orthodontic treatment.

Any treatment plan is a prediction of change. Due to the lack of visual treatment objectives, devising an IPO largely depends on the orthodontists’ subjective prediction of treatment results. The positioning of incisors is a problem that needs to be considered from many aspects, such as aesthetics improvement, function establishment and side-effect prevention. The devising of optimal incisor positions may differ among orthodontists, owing to their different background knowledge, aesthetic perception, and clinical experience.

The aim of this study was to introduce a new method of devising IPO on cephalograms and to test the intra-examiner reliability among orthodontists in the same group, as well as the inter-examiner reliability between different groups. The null hypothesis was that there would be no significant difference in the IPO measurements within each group over time, and there would be no significant difference in IPO measurements between different groups at each time point.

## Methods

The SmartOrtho software was used to visualize and quantify the orthodontic treatment objectives of incisors to facilitate the comparison among different orthodontists. The location of original incisors for each patient was identified and marked by a single orthodontist (author: Xue Li), and copies were distributed to all the examiners to ensure that different orthodontists start the IPO devising from the same location for each patient.

Permission to perform this study was approved by the Ethics Committee of the West China Hospital of Stomatology, Sichuan University (WCHSIRB-CT-2021-501). The pre-treatment lateral cephalograms (Veraviewepocs, Morita, Kyoto, Japan) of 60 patients (25 Class I, 32 Class II, and 3 Class III skeletal malocclusions according to the ANB angle, with an average age of 27.2 ± 6.4, range: 20–45 years) were included in the study. Inclusion criteria were: (1) Adult patients; (2) No missing anterior teeth before treatment; (3) Protrusive lips according to the E-line; and (4) Treatment involved four premolars extraction. Exclusion criteria were: (1) Severe facial asymmetry; (2) History of craniofacial defects or syndromes, e.g., cleft lip and palate; and (3) Severe skeletal discrepancy requiring orthognathic surgery.

**Fig. 1:**
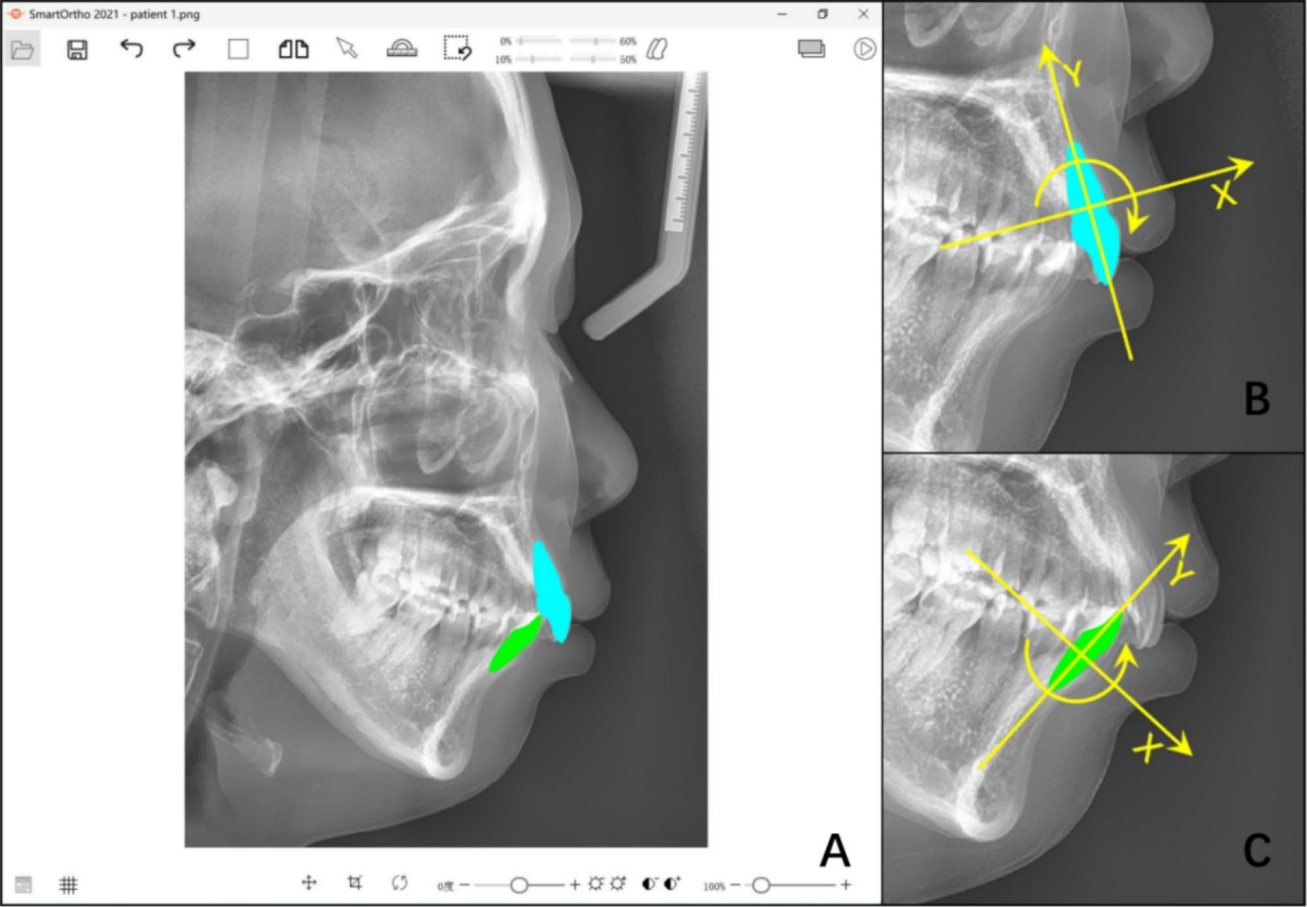
The incisor template and incisor movement in the SmartOrtho software. (**A**) The incisor templates presented in the SmartOrtho software. (**B**&**C**) Illustrations of the calculation of incisor movement: the incisor movement is decomposed into three types: the incisor torque movement (rotation around the origin, with lingual crown torque measured as positive value); the incisor protrusion/retraction movement (translation along the X-axis, with incisor retraction measured as positive value); and the incisor extrusion/intrusion movement (translation along the Y-axis, with incisor intrusion measured as positive value)

The cephalograms were imported into a cephalometric analysis software – the SmartOrtho (Sichuan University, Chengdu, China), and oriented according to the Frankfort plane. An incisor template and a rectangular coordinate system were generated by marking the original incisor’s incisal edge, labial cementum-enamel junction, lingual cementum-enamel junction and root apex (Fig. 1). The incisor template was allowed to rotate around the origin (simulating the incisor torque movement) and translate along the X-axis (simulating the incisor retraction/protrusion) and Y-axis (simulating the incisor extrusion/intrusion). The type and amount of incisor movement relative to the initial position could be directly read and exported by the SmartOrtho software.

Ten orthodontists were divided into to two groups based on their clinical experience: the junior group (N = 5) with clinical experience of less than 3 years, and the senior group (N = 5) with clinical experience of more than 10 years. Average duration of clinical experience for junior group is 2.4 ± 0.5 years, they were orthodontic residents who had more than 2 years of their residency program. The average duration of clinical experience for senior group is 11.0 ± 0.7 years, they were considered experts, at least 500 orthodontic patients were treated.

Before the formal experiment, all orthodontists practiced positioning/moving the incisor templates on another 10 different cephalograms to familiarise themselves with the SmartOrtho software. Subsequently, for the formal experiment, each orthodontist was asked to use the 60 included cephalograms and position the upper and lower incisor templates to the location where they thought optimal on the cephalograms, without any reference to the data of cephalometric analysis. The operation was repeated by the orthodontists again after a 2-week interval.

All statistical analyses were performed using the SPSS software (V23.0; IBM, Armonk, NY, USA). Paired *t*-test was used to evaluate the absolute difference between the first and second IPO devising of each group, and between the senior and junior group in each time’s IPO devising. Intraclass correlation coefficient (ICC) values were used to evaluate the inter-examiner and intra-examiner reliabilities, making use of the 2-way random model, average measures, absolute consistency type, and a 95% confidence interval.

## Results

The absolute differences between the first and second IPO devising of the two groups are shown in Table 1. There were significant differences in all types of upper incisor movement and lower incisor protrusion/retraction movement between the first and second IPO devising of the senior group. The junior group showed significant differences in the twice upper incisor extrusion/intrusion movement and upper incisor torque movement devising. The absolute differences between the two groups in each time’s IPO devising are shown in Table 2. There were significant differences in all types of incisor movement between the senior and junior groups in each time’s IPO devising.

**Table 1:** Absolute difference between the first and second IPO devising of each group and results of paired *t*-test

	T1 M (SD)	T2 M (SD)	Difference	F	P
**Senior group**					
U_P/R	3.83 (1.15)	3.98 (1.18)	-0.15 (0.82)	7.15	0.008*
U_E/I	4.18 (1.36)	4.36 (1.43)	-0.18 (0.95)	7.05	0.008*
U_T	6.07 (5.15)	5.10 (5.24)	0.97 (4.99)	1.11	0.293
L_P/R	1.71 (1.16)	1.83 (1.15)	-0.12 (0.77)	4.70	0.031*
L_E/I	2.73 (2.64)	2.51 (1.34)	0.22 (2.36)	1.83	0.177
L_T	10.69 (6.37)	10.69 (5.86)	-0.00 (5.33)	0.00	0.990
**Junior group**					
U_P/R	3.49 (1.52)	3.61 (1.67)	-0.12 (1.08)	1.80	0.180
U_E/I	3.33 (1.55)	3.12 (1.74)	0.20 (1.26)	4.61	0.032*
U_T	3.37 (6.39)	2.92 (5.20)	0.45 (3.97)	1.99	0.159
L_P/R	1.38 (1.38)	1.40 (1.52)	-0.03 (1.10)	0.07	0.797
L_E/I	2.26 (1.49)	2.33 (1.41)	-0.06 (1.19)	0.18	0.668
L_T	6.56 (5.81)	6.23 (5.84)	0.33 (4.27)	1.22	0.270

U_P/R: upper incisor protrusion/retraction movement; U_E/I: upper incisor extrusion/intrusion movement; U_T: upper incisor torque movement; L_P/R: lower incisor protrusion/retraction movement; L_E/I: lower incisor extrusion/intrusion movement; L_T: lower incisor torque movement

T1: the first IPO devising; T2: the second IPO devising; M: mean; SD: standard deviation

*: statistical significance at P<0.05

**Table 2:** Absolute difference between the two groups in each time’s IPO devising and results of paired *t*-test

	Senior group M (SD)	Junior group M (SD)	DF	Difference	T	P
**T1**						
U_P/R	3.79 (1.00)	3.45 (1.07)	59	0.34 (0.13)	3.514	0.000*
U_E/I	4.15 (1.19)	3.33 (1.24)	59	0.82 (0.62)	9.658	0.000*
U_T	5.95 (3.89)	3.46 (3.82)	59	2.49 (1.87)	10.420	0.000*
L_P/R	1.70 (0.91)	1.41 (1.13)	59	0.28 (0.75)	2.413	0.019*
L_E/I	2.70 (1.53)	2.29 (1.08)	59	0.41 (0.60)	3.565	0.001*
L_T	10.59 (5.01)	6.67 (4.99)	59	3.82 (2.73)	11.152	0.000*
**T2**						
U_P/R	3.98 (0.98)	3.55 (1.08)	59	0.43 (0.65)	4.575	0.000*
U_E/I	4.37 (1.18)	3.13 (1.26)	59	1.24 (0.68)	13.540	0.000*
U_T	5.08 (4.38)	2.97 (3.54)	59	2.11 (2.24)	7.062	0.000*
L_P/R	1.85 (0.94)	1.43 (1.03)	59	0.43 (0.70)	4.639	0.000*
L_E/I	2.53 (1.08)	2.22 (1.13)	59	0.31 (0.60)	2.538	0.014*
L_T	10.68 (5.01)	6.37 (4.90)	59	4.30 (2.52)	13.043	0.000*

T1: the first IPO devising; T2: the second IPO devising

M: mean; SD: standard deviation; DF: degrees of freedom; T: the t value of paired *t*-test

*: statistical significance at P<0.05

In the first IPO devising, the senior orthodontists had good consistency (ICC > 0.75) in all types of incisor movement devising except lower incisor extrusion/intrusion movement (ICC < 0.4). The junior orthodontists had good consistency in lower incisor extrusion/retraction movement and torque movement, and moderate consistency (0.4 < ICC < 0.75) in the other types of incisor movement. In the second IPO devising, the senior orthodontists exhibited poor consistency (ICC < 0.4) in lower incisor protrusion/retraction movement and good consistency in the other types of incisor movement. The junior orthodontists had good consistency in lower incisors extrusion/intrusion movement and torque movement, and moderate consistency in other types of incisor movement. Compared with the junior group, the senior group had lower mean absolute differences and higher agreement in all types of upper incisor movement in each time’s IPO devising. With respect to the lower incisor position objective devising, the junior group had lower mean absolute differences and higher agreement in lower incisor extrusion/intrusion and torque movement in the first IPO devising, and in the lower incisor protrusion/retraction movement in the second IPO devising (Table 3).

**Table 3:** Intra-examiner reliability

	T1 ICC (lower 95% CI, upper 95% CI)	P (T1)	T2 ICC (lower 95% CI, upper 95% CI)	P (T2)
**Senior group**				
U_P/R	0.872 (0.786,0.924)	0.000*	0.849 (0.749,0.910)	0.000*
U_E/I	0.892 (0.834,0.933)	0.000*	0.850 (0.705,0.918)	0.000*
U_T	0.777 (0.550,0.882)	0.000*	0.851 (0.775,0.906)	0.000*
L_P/R	0.806 (0.552,0.904)	0.000*	0.353(0.032,0.591)	0.000*
L_E/I	0.398 (0.127,0.608)	0.000*	0.814 (0.673,0.892)	0.000*
L_T	0.786 (0.547,0.889)	0.000*	0.886 (0.824,0.928)	0.000*
**Junior group**				
U_P/R	0.708 (0.472,0.835)	0.000*	0.620 (0.299,0.790)	0.000*
U_E/I	0.795 (0.682,0.872)	0.000*	0.729 (0.484,0.852)	0.000*
U_T	0.536 (0.154,0.745)	0.000*	0.645 (0.381,0.796)	0.000*
L_P/R	0.580 (0.376,0.731)	0.000*	0.629 (0.411,0.772)	0.000*
L_E/I	0.702 (0.531,0.816)	0.000*	0.808 (0.709,0.879)	0.000*
L_T	0.879 (0.816,0.924)	0.000*	0.875 (0.805,0.923)	0.000*

ICC: Intraclass correlation coefficient

T1: the first IPO devising; T2: the second IPO devising

*: p<0.05

As for the result of inter-examiner reliability, highest agreement and lowest mean absolute differences were found in lower incisor extrusion/intrusion movement in both IPO devising. The consistency of the two groups in the other types of incisor movement varied from moderate to good and all had high mean absolute differences (Table 4).

**Table 4:** Inter-examiner reliability

	T1 ICC (lower 95% CI, upper 95% CI)	P (T1)	T2 ICC (lower 95% CI, upper 95% CI)	P (T2)
U_P/R	0.783 (0.609,0.877)	0.000*	0.748 (0.476,0.869)	0.000*
U_E/I	0.712 (-0.008,0.898)	0.000*	0.565 (0.089,0.846)	0.000*
U_T	0.719 (-0.032,0.905)	0.000*	0.742 (0.207,0.894)	0.000*
L_P/R	0.709 (0.543,0.820)	0.000*	0.685 (0.384,0.832)	0.000*
L_E/I	0.811 (0.650,0.895)	0.000*	0.845 (0.738,0.909)	0.000*
L_T	0.644 (-0.073,0.874)	0.000*	0.632 (0.084,0.877)	0.000*

ICC: Intraclass correlation coefficient

T1: the first IPO devising; T2: the second IPO devising

*: p<0.05

## Discussion

High reliability relies on repeated measurements by the same or different examiners yielding consistent results [18]. In this study, we were concerned with the consistency of different orthodontists in IPO devising. As seen from the result of inter-examiner reliability, the senior and junior orthodontists were more likely to achieve an agreement on the lower incisor extrusion/intrusion movement. However, there was more variation in the devising of other types of incisor movement, which might be influenced by the orthodontists’ clinical experience.

Interestingly, during each time’s IPO devising, the senior orthodontists had high agreement and low mean absolute differences in upper incisor position objectives devising. It seemed that before IPO devising began, the senior orthodontists had a unified concept (ICC*: *p* < 0.05, which is defined as “there are no significant difference between the incisor positions made by orthodontists in the same group”, in other words, their concept and the results of IPO have kept step) in the upper incisor position objective devising. After the first IPO devising, this concept underwent some changes but remained unified. However, such a phenomenon did not appear in the junior orthodontists. They neither formed a unified concept before the first IPO devising nor after the first IPO devising. It can be inferred that with an increase in clinical experience, orthodontists were more likely to form a unified concept when devising the upper incisor position objectives, and this concept may change with the practice of IPO devising.

Although the study only included lip-protrusive patients, it is the most typical cases requiring IPO devising. As for clinical practice, orthodontists should combine other diagnostic materials, such as CBCT, panorama radiograph and study casts, to devise IPO more comprehensively [19, 20]. We suggest that the IPO devising method proposed in the present study be used as an auxiliary tool in orthodontic treatment planning, especially in digital teeth arrangement for clear aligner, customized labial and lingual orthodontic appliance treatment. The technician from the company can take the data of IPO, which devised and submitted by the orthodontists, as reference for incisors and other teeth arrangement. It is expected to improve the accuracy and efficiency of digital teeth arrangement.

The significance of this article, in addition to comparing the design reliability of the incisor target position of doctors in two ages, could also be concluded as follows. First, this model can be further promoted. This study can show that for young doctors, the superior doctors need to pay attention to the incisor position design and other problems during supervision. Of course, older doctors may prefer more adduction and more lingual inclination, which could provide valuable reference for increasing the accuracy of the IPO design. Second, it can be further studied whether there are differences in the aesthetics of doctors in the two ages, such as the era and environment of contact, education, etc., whether the aesthetics [21] formed in this way will be one of the factors affecting the design of IPO? References can be found on aesthetic among orthodontists, or similar age-related design factors [21]. It is equivalent to that our research can provide reference ideas for subsequent research, so the significance of this research can be imagined as a relatively important “key”. Third, this idea of testing inter- and intra-examiner reliability via a software was novel and convenient, for that deciding the IPO on a 2D model was more visualized. Herein, this research could also provide reference for the future IPO design among orthodontists of different genders, districts or with education backgrounds.

While the study focused on lip-protrusive patients, the IPO devising method can be a valuable addition to orthodontic treatment planning when combined with other diagnostic materials, and it has the potential to improve the precision and effectiveness of digital teeth arrangement.

## Conclusion

The senior and junior orthodontists have the best inter-examiner reliability in lower incisor extrusion/intrusion movement. The senior orthodontists had better intra-examiner reliability in upper incisor position objectives devising than the junior orthodontists. The senior orthodontists tended to devise the incisor more recessive, more intrusive, and more lingual crown torque than the junior orthodontists.

## Data Availability

The datasets used and analysed during the current study are available from the corresponding author on reasonable request.

## Abbreviations

FMA: Frankfort mandibular angle
FMIA: Frankfort mandibular incisor angle
ICC: Intraclass correlation coefficient
IPO: Incisor position objective
